# Supplementary material for: Chromothripsis during telomere crisis is independent of NHEJ, and consistent with a replicative origin
Source: Genome Res. 2019 May;29(5):737–49. doi: 10.1101/gr.240705.118 (PMC6499312; doi:10.1101/gr.240705.118)
Supplement: Supplemental Material [file supp_gr.240705.118_Supplemental_file_1.zip › contigs/annotated_contigs/DB47/contig.2.DB47_length_621_mean_cov_7.97101449275.docx]

**DB47_length_621_mean_cov_7.97101449275**

```
GCATGGTTAAGGTTGATGTGGACCATGTCCATGGCCTCCGTCTTCTCTGGAAACTGGAGGCTGGTGGCTGCAGGTGGTCCATGCCCTTC
 >chr4:14688244-14688550 + E=2e-172
CCCTGCTAAAGTCATTTGATGAAAAGGAAGGTGATTTAAAGATATAGCCCTAGTAATTCGCTTTTCCAATACGTAATTGGTATTTTAGA

GCATCATAAAGTTATTGGTCTCTAAAACTGTGCCTTTCTCAGAATGTTTTAGGTGAGTTCTACTTTTCTGCCAGTCCACTTTTAAATCT

TGTTTTGTCAATTATTTCTCTTCTGTATACTTTACG|TTG|GCTGAACCACTCACTATTTTAGGTGGTTGGTACAAGTGAGTGAAGGTC
                                     >chr4:14691433-14691751 + E=1e-179
AGGAGCTCTTGACTAGAGTAGCAGCAATTTTACTTAGATTTACATTTATTATTGAACATGGGTCATACTCTCTAATATCTGTGCTAAAT

AGTAGCTGTTGTTGAATATTAATAATCTCTATTATCACTGTGAAGTCTGCAATAGGCTTTAAAATAATGCACACAGACATATAAATCTA

TGAATATATTTCTCACTTTCCTTCAAAGGGGCCCATTGTAATTTGCGTACTCTACATCAGGATTCTTTTTGTCCAGTGTTCCTCAGCTT
```
